# Utilization of zinc chloride for surface modification of activated carbon derived from *Jatropha curcas* L. for absorbent material

**DOI:** 10.1016/j.dib.2016.11.019

**Published:** 2016-11-13

**Authors:** P. Pratumpong, S. Toommee

**Affiliations:** a Department of Physics, Faculty of Science and Technology, Thammasat University, Patumtani 12120, Thailand; b Industrial Product Design and Development Program, Faculty of Industrial Technology, Kamphaeng Phet Rajabhat University, Kamphaeng Phet 62000, Thailand

**Keywords:** Activated carbon, *Jatropha curcasL.*, Porous materials

## Abstract

The objective of this research is to produce the low-cost activated carbon from *Jatropha curcas L.* by chemical activation using zinc chloride ZnCl_2_. The effects of the impregnation ratio on the surface and chemical properties of activated carbon were investigated. The impregnation ratio was selected at the range of 1:1–10:1 for investigation. The optimum conditions resulted in an activated carbon with a carbon content of 80% wt, while the specific surface area evaluated using nitrogen adsorption isotherm corresponds to 600 m^2^/g.

**Specifications Table**

**Table:** 

Subject area	*Materials*
More specific subject area	*Activate carbon*
Type of data	*Table, Figure*
How data was acquired	*FTIR, SEM, adsorption efficiency*
Data format	*Analyzed*
Experimental factors	*Surface modification of activated carbon from Jatropha curcas L. waste by zinc chloride treatment*
Experimental features	*The objective of this research is to produce the low-cost activated carbon from Jatropha curcas by chemical activation using zinc chloride. The effects of the impregnation ratio on the surface and chemical properties of activated carbon were investigated. The impregnation ratio was selected at the range of 1:1–10:1 for investigation. The optimum conditions resulted in an activated carbon with a carbon content of 80% wt, while the specific surface area evaluated using nitrogen adsorption isotherm corresponds to 600 m*^*2*^*/g.*
Data source location	*Faculty of Science and Technology, Thammasat University, Thailand*
Data accessibility	*Data are provided in this article*

**Value of the data**

• Activated carbon was prepared from *Jatropha curcas*.
• Pyrolysis technique was employed to prepare activated carbon.
• Activated carbon can be employed as sensor material, membrane technology and catalysis materials.

## 1 Data

Data of the as-synthesized activated carbon from Jatropha curcas.

## 2 Experimental design, materials and methods

Table 1 exhibits elemental analysis of activated carbon after surface modification on conventional reaction of zinc chloride [1], [2], [3]. The percent yield of activated carbon after zinc chloride modification was estimated to be 40–48 wt %.

**Fig. 1:**
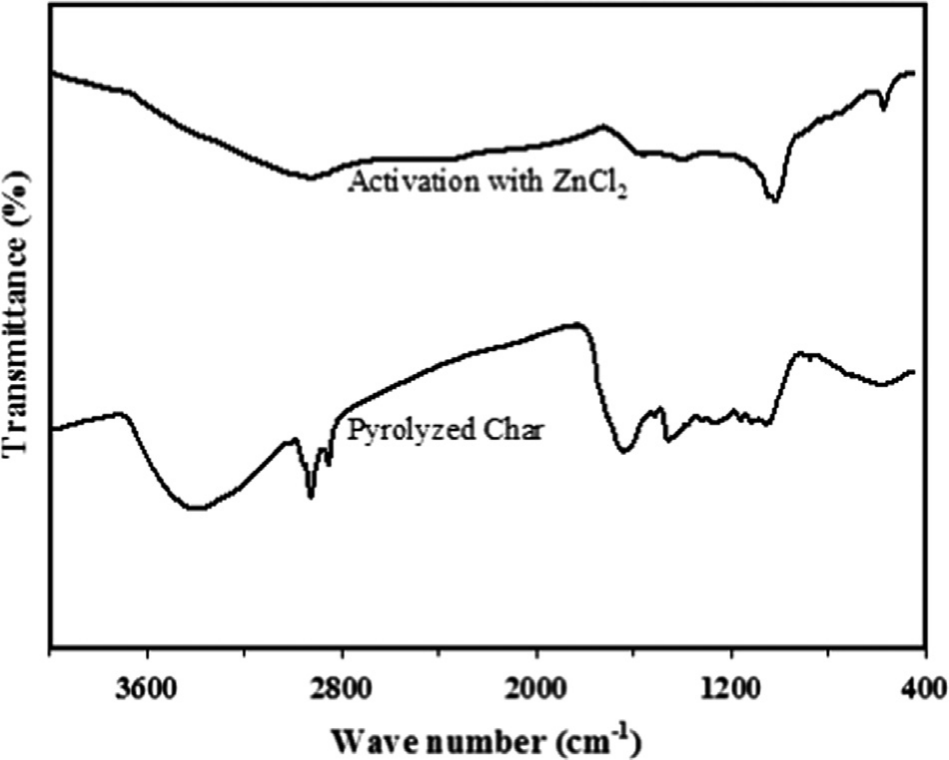
FTIR spectra of surface modification of activated carbon derived from *Jatropha curcas*.

Fig. 1 shows that the functional groups of activated carbon differ significantly from those of pyrolyzed char [4], [5], [6], [7], [8]. The spectrum from char at 3393 cm^−1^ indicated the presence of the –OH group of phenol. The methylene group is detected by –CH stretching at a wave number of 2924 cm^−1^. The aldehyde group of –O–CH_3_ is found around 2853 cm^−1^. Strong bands at 1641 cm^−1^ indicate C–O stretching of carboxyl or carbonyl groups. Methyl or amine groups are shown by a peak around 1385–1380 cm^−1^. The band from 1200 to 1000 cm^−1^ is the fingerprint of syringyl units. Aldehyde and derivatives of benzene are detected by peaks at 875 and 761 cm^−1^.

**Fig. 2:**
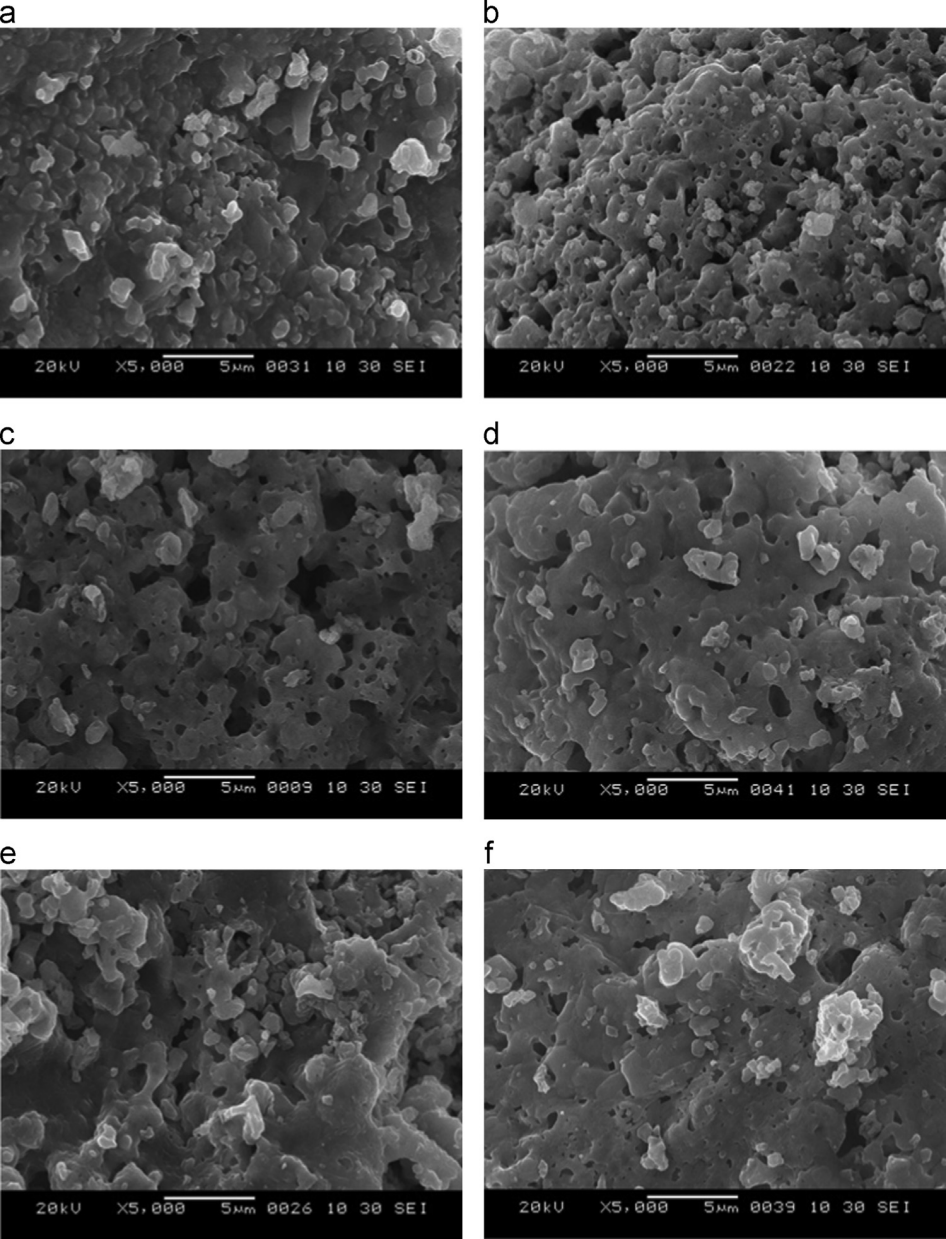
Morphological properties of activated carbon and its surface modification with reaction of zinc chloride (a) activated carbon (b) AC 1:1 (c) AC 3:1 (d) AC 5:1 (e) AC 7:1 (f) AC 10:1.

Fig. 2 exhibits the morphological properties of activated carbon derived from *J. curcas* and its surface modification by zinc chloride. Without any surface modification, the porous structure was less. It exhibited the non-uniform structure of agglomerated particle.

**Fig. 3:**
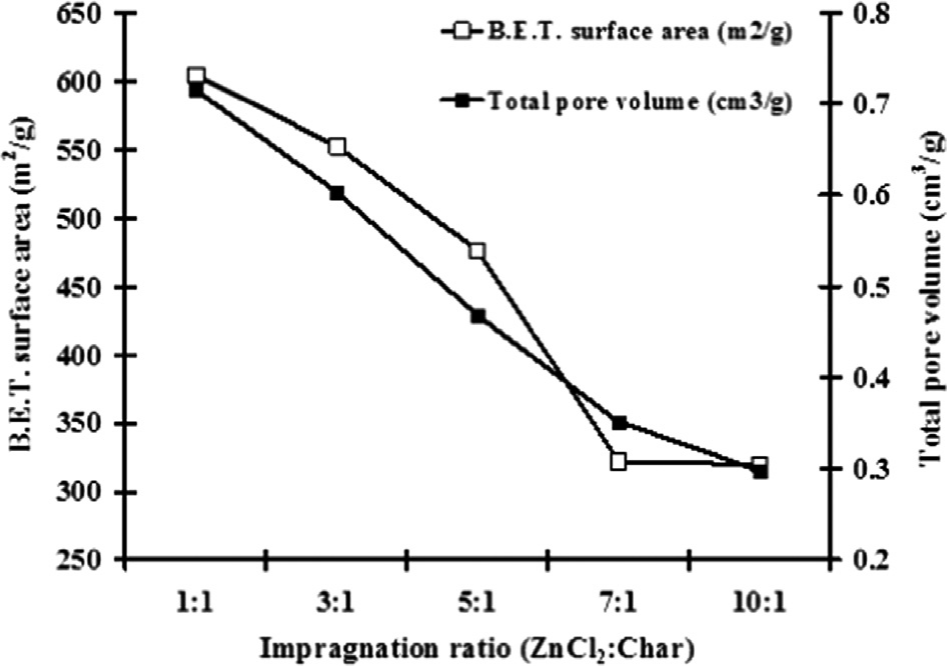
Nitrogen adsorption isotherm of produced activated carbon modified by zinc chloride.

Fig. 3 exhibits the N_2_ adsorption isotherm of activated carbon derived from *J. curcas*. It was important to note that the specific surface area, pores size and pore volume were increased with respect to impregnation ratio from 1.0 to 10.0. The maximum specific surface area was due to 604.31 m^2^/g.

**Fig. 4:**
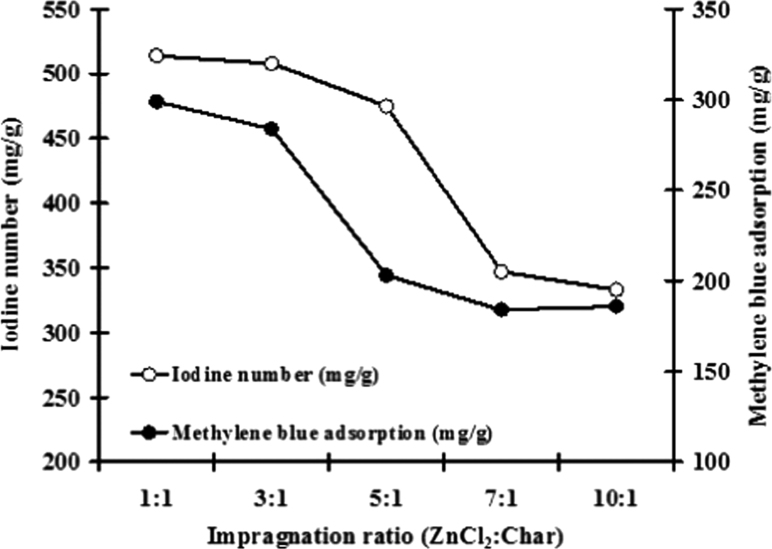
Analytical data on iodine number and methylene blue adsorption.

Fig. 4 exhibits the iodine number and methylene blue adsorption of activated carbon. When the activating agent comes in contact with the char, it reacts both with the exterior and the interior of the particle, in which most of the disorganized carbon is removed. With regard to the effect of impregnation ratio, the result indicated iodine number and methylene blue adsorption range of 333–514 and 186–299 mg/g, respectively.

## Figures and Tables

**Table 1:** Elemental analysis of activated carbon after chemical modification.

Sample	Impregnation ratio	Elemental analysis (wt%)				Yield (wt%)
		C	H	N	O^a^	
Physic nut waste char	–	70.70	6.20	1.00	22.80	36.45
AC_1:1_	1:1	83.10	5.50	3.40	8.00	47.82
AC_3:1_	3:1	80.40	5.80	2.50	11.30	45.30
AC_5:1_	5:1	78.30	6.40	2.60	12.70	44.50
AC_7:1_	7:1	75.70	6.90	2.10	15.30	42.27
AC_10:1_	10:1	71.50	6.00	1.80	20.70	40.09

a By different.
