# Supplementary material for: Utilization of zinc chloride for surface modification of activated carbon derived from Jatropha curcas L. for absorbent material
Source: Data Brief. 2016 Nov 13;9:970–5. doi: 10.1016/j.dib.2016.11.019 (PMC5121167; doi:10.1016/j.dib.2016.11.019)
Supplement: Supplementary file 1 — Supplementary material [file mmc1.docx]

Conflict of Interest

There is no conflict of interest for this manuscript submitted to Data in Brief.
